# Supplementary material for: A Scoping Review of Capacity-Building Efforts to Address Environmental Justice Concerns
Source: Int J Environ Res Public Health. 2020 May 26;17(11):3765. doi: 10.3390/ijerph17113765 (PMC7312702; doi:10.3390/ijerph17113765)
Supplement: Supplementary file 1 [file ijerph-17-03765-s001.pdf]

**Table S1.** Systematic Search Strategy.

| **Key Terms Applied Across Databases** | | |
|---|---|---|
| Community Based<br>Community Capacity<br>Community Engaged<br>Community Intervention<br>Community Organizing | Environmental health<br>Environmental justice AND (discrimination, equity, inequality, or racism)<br>Environmental policy<br>Environmental racism | Health disparity (disparities)<br>Low income<br>Minority<br>Program evaluation<br>Theory AND (program, intervention, or project) |

AND

| Environment Complete | Political Science Complete | PubMed | SocIndex | Web of Science |
|---|---|---|---|---|
| Environment Protection Planning<br>Environmental Change<br>Neighborhood Empowerment<br>Policy Change<br>Policy Reform<br>Policy Solution | Citizen participation<br>Community-engaged<br>Grassroots activism<br>People of color<br>Policy change<br>Policy outcomes<br>Policy solution<br>Political participation<br>Social movement | Community effort<br>Community empowerment<br>Community mobilization<br>Community of color<br>Environmental intervention<br>Environmental policy change<br>Organizing communities<br>Policy outcomes<br>Systems change | Community engaged<br>Mobilizing communities<br>environmental change intervention<br>Organizing communities<br>Policy outcome<br>Policy solution<br>Systems change | Community Empowerment<br>Community evaluation<br>Community study<br>Environmental intervention<br>Policy change |

**Table S2.** Included Articles for Systematic Scoping Review.

| | |
|---|---|
| *Allen, B. L. (2001). "Saving St. Gabriel: The Emergence of a New African American town." Contemp Justice Rev 4(2): 145.* | Authors detail a case study of St. Gabriel, a predominantly Afro-American town in Louisiana and analyzes community change via social justice activism. |
| *Allen, M., et al. (2017). "Stronger together: Strategies to protect local sovereignty, ecosystems, and place-based communities from the global fossil fuel trade." Marine Policy 80: 168-176.* | This paper examines how a community has collaborated and succeeding in blocking construction of the coal-shipping port Gateway Pacific Terminal (GPT) |
| *Balazs, C. L. and R. Morello-Frosch (2013). "The Three Rs: How Community-Based Participatory Research Strengthens the Rigor, Relevance, and Reach of Science." Environ Justice (19394071) 6(1): 9-16.* | This research focuses on environmental health CBPR methods used in two case study examples from the Northern California Household Exposure Study, and the San Joaquin Valley Drinking Water Study. |
| *Barry, J. M. (2008). ""A Small Group of Thoughtful, Committed Citizens": Women's Activism, Environmental Justice, and the Coal River Mountain Watch." Environ Justice (19394071) 1(1): 25-33.* | This article details a case study analysis of the environmental justice efforts of the West Virginia Coal River Mountain Watch organization. |
| *Bell, S. E. (2015). "Bridging Activism and the Academy: Exposing Environmental Injustices Through the Feminist Ethnographic Method of Photovoice." Human Ecol Rev 21(1): 27-58.* | The authors present a photovoice project conducted among women living in five coal-mining communities in southern West Virginia. |
| *Blodgett, A. D. (2006). "An Analysis of Pollution and Community Advocacy in 'Cancer Alley': Setting an Example for the Environmental Justice Movement in St James Parish, Louisiana." Local Environ 11(6): 647-661.* | This study evaluates environmental justice issues in St James Parish, Louisiana, details the use of geographic information system mapping to identify locations of polluting industries, and identifies community advocacy strategies in response to environmental impacts. |
| *Brickle, M. B. and R. Evans-Agnew (2017). "Photovoice and Youth Empowerment in Environmental Justice Research: A Pilot Study Examining Woodsmoke Pollution in a Pacific Northwest Community." J Community Health Nurs 34(2): 89-101.* | The authors examined youth empowerment in a citizen science study on woodsmoke, using Photovoice methodology. |
| *Brown, P. and S. Masterson-Allen (1994). "The Toxic-waste movement – A new Type of Activism." Soc Nat Resour 7(3): 269-287.* | The authors discuss the toxic waste movement, the role of activism, the mobilization process, and the new global movement as an approach to address environmental justice advocacy. |
| *Brown, P., et al. (2003). "The Health Politics of Asthma: Environmental Justice and Collective Illness Experience in the United States." Soc Sci Med 57(3): 453-464.* | This study investigates the role of outdoor environmental factors in causing or triggering asthma and compares the community organizing efforts of two community environmental justice organizations to address asthma in poor and minority communities. |
| *Bruno, T. and W. Jepson (2018). "Marketisation of Environmental Justice: U.S. EPA Environmental Justice Showcase Communities Project in Port Arthur, Texas." Local Environ 23(3): 276-292.* | This paper focuses on activities, objectives, and processes of intervention related to the state of Texas response to persistent claims of environmental injustice among residents of the overburdened community of Port Arthur. |
| *Bullard, R. D. and B. H. Wright (1990). "The Quest for Environmental Equity-Mobilizing the African American community for social change." Soc Nat Resour 3(4): 301-311.* | The author discusses several case studies across the Southern United States as examples of environmental injustice and details opposition strategies implemented by grassroots leaders and activists to redress their environmental problems. |
| *Capek, S. M. (1992). "Environmental Justice, Regulation, and the Local-community." Int J Health Serv 22(4): 729-746.* | This article examines the mobilization patterns in the city of Jacksonville, Arkansas, discusses the US EPA's Technical Assistance Grants program, and explores the concept of community in relation to grassroots organizations fighting against contamination. |

| | |
|---|---|
| *Čapek, S. M. (1993). "The "Environmental Justice" Frame: A Conceptual Discussion and an Application." Soc Problems 40(1): 5-24.* | The author discusses the dimensions of the environmental justice framework within the context of a case study of an African-American community in Texarkana, Texas that successfully mobilized for the declaration of their community as a Superfund site and federal buyout |
| *Cohen, A. K., et al. (2016). "Surveying for Environmental Health Justice: Community Organizing Applications of Community-Based Participatory Research." Environ Justice 9(5): 129-136.* | This study presents findings from a CBPR-based environmental health survey that quantified neighborhood concerns and health problems, conducted by a team of community organizers and academic researchers in Richmond, CA. |
| *Corburn, J. (2002). "Combining Community-based Research and Local Knowledge to Confront Asthma and Subsistence-fishing Hazards in Greenpoint/Williamsburg, Brooklyn, New York." Environ Health Perspect 110 Suppl 2: 241-248.* | In a case study review, the author highlights the work of two community-based organizations that have engaged in CBPR to address asthma and risks from subsistence-fish diets. |
| *Dhillon, C. M. (2017). "Using Citizen Science in Environmental Justice: Participation and Decision-making in a Southern California Waste Facility Siting Conflict." Local Environ 22(12): 1479-1496.* | This qualitative research analyses the relationship between citizen science and EJ in a new waste facility siting conflict in urban Los Angeles. |
| *Dressel, A., et al. (2013). "The Westlawn Partnership for a Healthier Environment: Promoting Environmental Justice and Building Community Capacity." Environ Justice (19394071) 6(4): 127-132.* | This study is an evaluation of the US EPA Community Action for a Renewed Environment (CARE) co-operative agreements between public health professionals, business leaders, K-16 schools, non-profit organizations, community residents, and other community stakeholders who formed a coalition to reduce toxics in the neighborhood. |
| *Drury, R. T. (2008). "Moving a Mountain: The Struggle for Environmental Justice in Southeast Los Angeles." Environ Law Reporter: News & Analysis 38(5): 10338-10346.* | This case study demonstrates the power of community organizing, scientific research, and common law in a public nuisance suit against a concrete-crushing facility in southeastern Los Angeles County. |
| *Emmett, E. A. and C. Desai (2010). "Community First Communication: Reversing Information Disparities to Achieve Environmental Justice." Environ Justice 3(3): 79-84.* | This study details the application of CBPR as a strategy to reverse long-standing information disparities, empower a community, and provide motivation for behavioral change by industry and residents. |
| *Garcia, A. P., et al. (2013). "THE (Trade, Health, Environment) Impact Project: A Community-Based Participatory Research Environmental Justice Case Study." Environ Justice (19394071) 6(1): 17-26.* | In this multi-method case study analysis, the authors describe the informal collaborative work which culminated in a regional CBPR partnership between two universities and four community-based advocacy groups to address air pollution and other health impacts associated with goods movement through the massive Los Angeles and Long Beach Ports complex. |
| *Gonzalez, P. A., et al. (2011). "Community-Based Participatory Research and Policy Advocacy to Reduce Diesel Exposure in West Oakland, California." Am J Public Health 101: S166-S175.* | In this study the authors conducted a multimethod case study analysis of a CBPR partnership in West Oakland, California, and its efforts to study and address the neighborhood's disproportionate exposure to diesel air pollution. |
| *Green, L., et al. (2002). ""Hey, Mom, Thanks!": Use of Focus Groups in the Development of Place-specific Materials for a Community Environmental action campaign." Environ Health Perspect 110 Suppl 2: 265-269.* | This study discusses the relevancy of five strategies to reduce the risk of exposure to environmental hazards for African-American and Hispanic children living in Northern Manhattan in New York City. |
| *Grineski, S. E. (2006). "Local Struggles for Environmental Justice: Activating Knowledge for Change." J Poverty 10(3): 25-49.* | This paper is an ethnography of a CBPR effort for environmental justice in Phoenix, Arizona that analyses how neighborhood struggles can result in socioenvironmental change. |

| | |
|---|---|
| *Haynes, E. N., et al. (2011). "Developing a Bidirectional Academic-Community Partnership with an Appalachian-American Community for Environmental Health Research and Risk Communication." Environ Health Perspectives 119(10): 1364-1372.* | This study details an academic partnership with an Appalachian-American community that implemented CBPR principles to develop a community driven research agenda to address air quality concerns. |
| *Higman, K., et al. (2007). "Using the PACE EH Model to Mobilize Communities to Address Local Environmental Health Issues - A Case Study in Island county, Washington." J Environ Health 70(1): 37-41.* | This paper describes the methodology utilized by the the Island County Environmental Health Initiative to address locally important environmental health issues so that other local and state environmental health agencies may replicate the process in their communities. |
| *Hines, R. I. (2015). "The Price of Pollution: The Struggle for Environmental Justice in Mossville, Louisiana." Western J Black Studies 39(3): 198-208.* | This study chronicles the historic disproportionate burden of industrial pollution and environmental struggle as well as the power of community empowerment and mobilization among residents of Mossville, Louisiana. |
| *Jacobs, J. (1992). "A Community Organizing Case Study: An Analysis of Cap-It's Strategy to Prevent the Location of a Toxic Waste Incinerator in Their Community." Int Q Community Health Educ 13(3): 253-263.* | This case study profiles a collection of middle-class residents living in a small working-class town and their successful battle to prevent the siting of a hazardous waste incinerator. |
| *Jiao, Y., et al. (2015). "Application of Citizen Science Risk Communication Tools in a Vulnerable Urban Community." Int J Environ Res Public Health 13(1): ijerph13010011.* | The authors detail the development of a community-led coalition and subsequent application of public participatory geographical information systems to design and conduct soil sampling for residents of Stambaugh-Elwood (SE) community in Columbus, OH. SE community. |
| *Johnson, G. S. (2005). "Grassroots Activism in Louisiana." Humanity Soc 29(3/4): 285-304.* | This article presents a case study on grassroots activism, using examples of two parish towns of Louisiana identified as sacrifice zones. |
| *Kaufman, A., et al. (2017). "A Citizen Science and Government Collaboration: Developing Tools to Facilitate Community Air Monitoring." Environ Justice 10(2): 51-61.* | This pilot study highlights the collaboration of EPA Region 2 and the Ironbound Community Corporation (ICC) in Newark, New Jersey, in assessing the utility of a citizen science air quality toolbox to address environmental concerns within the community. |
| *Kaup, B. Z. and D. Casey (2016). "Coalition of injustice? Bodies, business, and the biosphere in struggles against unwanted land uses." Environ Politics 25(3): 494-512.* | This article examines how the complex geographies of environmental injustice play out in a coalition to prevent the construction of a coal-fired power plant in Surry County, Virginia. |
| *Kegler, M. C., et al. (2010). "Primary Prevention of Lead Poisoning in Rural Native American Children Behavioral Outcomes from a Community-Based Intervention in a Former Mining Region." Fam Community Health 33(1): 32-43.* | The current study examined the effectiveness of a community-based lay health advisor intervention, combined with youth engagement, in improving lead poisoning prevention behaviors and associated beliefs in a rural Native American population located in and near a Superfund site containing mining waste. |
| *Kreger, M., et al. (2011). "Creating An Environmental Justice Framework for Policy Change in Childhood Asthma: A Grassroots to Treetops Approach." Am J Public Health 101 Suppl 1: S208-216.* | The authors detail the work of a network of coalitions, policy advocates, and technical assistance providers in California using an environmental justice approach to reduce risk factors for asthma in school-aged children. |
| *Loh, P., et al. (2002). "From Asthma to AirBeat." Environ Health Perspect 110: 297.* | This article discusses the CBPR project AirBeat that was designed to answer community questions about whether there are pollution "hot spots" in Roxbury and the degree to which diesel emissions are contributing to health problems. |
| *Miller, P. K., et al. (2013). "Community-based Participatory Research Projects and Policy Engagement to Protect Environmental Health on St Lawrence Island, Alaska." Int J Circumpolar Health 72.* | This article synthesizes discussion of collaborative research results, interventions and policy engagement for St Lawrence Island (SLI), Alaska, investigating levels of polychlorinated biphenyls (PCBs) at an abandoned military site and subsequent impact on local foods. |

| | |
|---|---|
| *Minkler, M. (2010). "Linking Science and Policy Through Community-Based Participatory Research to Study and Address Health Disparities." Am J Public Health 100: S81-S87.* | The authors use two case studies to highlight the role of CBPR in helping achieve policy changes that promote access to healthy foods (Bayview, San Francisco, CA) and higher air quality standards (Harlem, New York, NY). |
| *Minkler, M., et al. (2010). "Si Se Puede: Using Participatory Research to Promote Environmental Justice in a Latino community in San Diego, California." J Urban Health 87(5): 796-812.* | This paper explores a successful CBPR and organizing effort, the Toxic Free Neighborhoods Campaign, in Old Town National City, CA, and its contributions to both local policy outcomes and changes in the broader policy environment. |
| *Parker, N. E. A., et al. (2010). "Community Organizing Network for Environmental Health: Using A Community Health Development Approach to Increase Community Capacity Around Reduction of Environmental Triggers." J Prim Prev 31(1-2): 41-58.* | The article is an evaluation of the Community Organizing Network for Environmental Health project and identifies the dimensions of community capacity that were enhanced as a result of the CBPR efforts to reduce environmental triggers associated with childhood asthma. |
| *Robinson, E. E. (2016). "Sharing Stories." Humanity Soc 40(4): 442-461.* | The focus of this study is the role that health narratives play in mobilization when shared among community members experiencing exposure to air pollution from numerous sources of industrial pollution. |
| *Rohlman, D., et al. (2015). "A Community-Based Approach to Developing a Mobile Device for Measuring Ambient Air Exposure, Location, and Respiratory Health." Environ Justice 8(4): 126-134.* | In this study the authors use focus groups to develop and test a prototype mobile device to measure personal chemical exposure, location, and respiratory function. |
| *Sadd, J., et al. (2014). "The Truth, the Whole Truth, and Nothing but the Ground-Truth: Methods to Advance Environmental Justice and Researcher-Community Partnerships." Health Educ Behav 41(3): 281-290.* | This study documents the implementation of an environmental justice screening methodology in which community members worked with researchers to collect data across six Los Angeles neighborhoods to identify the clustering of potentially hazardous facilities, high levels of air pollution, and elevated health risks. |
| *Sanchez, H. K., et al. (2017). "Confronting Power and Environmental Injustice: Legacy Pollution and the Timber Industry in Southern Mississippi." Soc Nat Resour 30(3): 347-361.* | This case study uses an environmental justice framework to understand the historic pollution and community response related to a decommissioned creosote facility in Southern Mississippi. |
| *Schelly, D. and P. B. Stretesky (2009). "An Analysis of the "Path of Least Resistance" Argument in Three Environmental Justice Success Cases." Soc Nat Resour 22(4): 369-380.* | In this study, the authors detail three community-based environmental justice groups that have succeeded in stopping the siting of an industrial facility. |
| *Schwartz, N. A., et al. (2015). ""Where They (live, work and) Spray": Pesticide Exposure, Childhood Asthma and Environmental Justice Among Mexican-American Farmworkers." Health Place 32: 83-92.* | This article details an exploratory ethnographic research approach of photovoice used to study the relationship between air quality, childhood exposure to pesticide, and asthma. |
| *Senier, L., et al. (2008). "Brown Superfund Basic Research Program: A Multistakeholder Partnership Addresses Real-World Problems in Contaminated Communities." Environ Sci Tech 42(13): 4655-4662.* | This paper reports on a project by the Brown University Superfund Basic Research Program and details a case study involving community engagement, research translation, and collaboration with state regulatory agency personnel to mitigate the financial impacts of living in a contaminated community. |
| *Sherman, D. J. (2008). "Disruption or Convention? A Process-based Explanation of Divergent Repertoires of Contention Among Opponents to Low-level Radioactive Waste Disposal Sites." Soc Movement Stud 7(3): 265-280.* | In this case study approach, the author discusses two rural New York State county examples that each faced a state-imposed low-level radioactive waste site proposal and launched vigorous collective opposition, citizen lobbying and litigation efforts to resist the proposed facilities. |
| *Sicotte, D. (2010). "Don't Waste Us: Environmental Justice through Community Participation in Urban Planning." Environ Justice 3(1): 7-11.* | This article examines environmental justice lessons from the Eastwick community, a group of 11 racially diverse urban neighborhoods in industrial Southwest Philadelphia, Pennsylvania, USA. |

| | |
|---|---|
| *Spencer-Hwang, R., et al. (2016). "Strategic Partnerships for Change in an Environmental Justice Community: The ENRRICH Study." Prog Community Health Partnersh 10(4): 541-550.* | This paper shares lessons learned from a partnership with an elementary school, university researchers, and a local community-based organization to develop interventions and mitigation plans to reduce adverse child health impacts related to the close proximity of the San Bernardino Railyard. |
| *Stedman-Smith, M., et al. (2012). "Photovoice in the Red River Basin of the north: a systematic evaluation of a community-academic partnership." Health Promot Pract 13(5): 599-607.* | In this study, the authors detail an evaluation of community-academic partnership that used community photovoice methods to document pathways to pesticide exposure and other health and safety concerns of local children living in the Red River Basin, Minnesota. |
| *Sullivan, J. and J. Parras (2008). "Environmental Justice and Augusto Boal's Theatre of the Oppressed: a Unique Community Tool for Outreach, Communication, Education and Advocacy." Theory in Action 1(2): 20-39.* | In this article, the authors describe the application of The Forum Theater as a transformative tool to promote scientific literacy and deconstruct the complexities of how environmental injustices adversely affect both physical and mental health, and the larger economy of impacted communities. |
| *Tajik, M. and M. Minkler (2006). "Environmental justice research and action: a case study in political economy and community-academic collaboration." Int Q Community Health Educ 26(3): 213-231.* | This article describes a case study of community-university partnership among the Concerned Citizens of Tillery, and researchers at the University of North Carolina, Chapel Hill, School of Public Health to study and curb the growth of industrial hog operations and their adverse health effects in the United States' rural south. |
| *White, B. M. and E. S. Hall (2015). "Perceptions of environmental health risks among residents in the "Toxic Doughnut": opportunities for risk screening and community mobilization." BMC Public Health 15: 1230.* | In this study, the authors detail the groundwork for a community-based environmental health assessment, the development of a questionnaire, and implementation of focus groups to assess environmental health risk issues impacting the Altgeld Gardens, Chicago residents |
| *Wier, M., et al. (2009). "Health, Traffic, and Environmental Justice: Collaborative Research and Community Action in San Francisco, California." Am J Public Health 99 Suppl 3: S499-504.* | The authors of this study detail research assessing the effects of the construction of an intraurban freeway on residents of the Excelsior neighborhood in southeast San Francisco to facilitate community education and action to address transportation-related health burdens on neighborhood residents. |
| *Williams, E. M., et al. (2009). "Behind the Fence Forum Theater: An Arts Performance Partnership to Address Lupus and Environmental Justice." New Solut 19(4): 467-479.* | This article discusses the Buffalo Lupus Project, a CBPR partnership formed to explore the relationship between a local waste site and high rates of lupus and the use of performance theater. |
| *Wilson, S., et al. (2017). "Soil Contamination in Urban Communities Impacted by Industrial Pollution and Goods Movement Activities." Environ Justice 10(1): 16-22.* | In this study, the authors detail a community-university partnership and application of citizen science methods to assess baseline pollution levels of trace metals in soil near industrial pollution sources and heavily trafficked roadways in Charleston. |
| *Wilson, S. M., et al. (2007). "Use of EPA Collaborative Problem-solving Model to Obtain Environmental Justice in North Carolina." Prog Community Health Partnersh 1(4): 327-337.* | This paper highlights the work of the West End Revitalization Association, a community-based organization in Mebane, North Carolina, and their efforts to bring stakeholders in three low-income African-American communities together for collaboration. |
| *Wing, S., et al. (2008). "Integrating Epidemiology, Education, and Organizing for Environmental Justice: Community Health Effects of Industrial Hog Operations." Am J Public Health 98(8): 1390-1397.* | In this article, the authors investigate the relationships between the resulting pollution and the health and quality of life of the hog operations' neighbors; and is designed to link research with community education and organizing for social justice in eastern North Carolina. |

**Table S3.** Content Abstraction Form.

**Introduction**

This form is for screening full-text articles for inclusion in the systematic search and scoping review of community problem solving efforts to address environmental problems.

Community is defined as a collective effort by a group of people to eliminate a point source of pollution affecting their community

Please use the comments area to note any problems you had extracting these data (including where there was not enough information in the paper to answer fully)

**Important considerations:**

- English language only
- Peer reviewed academic journal articles only (excludes grey literature, conference abstracts, book reviews)
- Publication discusses community organizing actions since 1986
- Work must be completed and not just planned (excludes protocol registration and cohort announcements)
- Exclude articles that: do not engage with community (e.g. school-based only research), focus on physical activity, nutrition (organic food advocacy), greenspace, tobacco.

**Reviewer: __________Article Number: _______Author Last Name: _____________Publication Year: _________**

☐ **Article excluded for further review**

**Reason for exclusion:**

☐ Conference abstract, book review, systematic review or other literature review

☐ Summary report or government document

☐ Grey literature: newspaper, magazine, website, summary report

☐ Research is not focused on pollution

☐ Research does not directly engage with community

☐ Research is not completed

☐ Other: __________________________

---

**I. Author affiliation/discipline (captures affiliations of all contribution authors; check all that apply)**

☐ Environmental discipline

☐ Other public health discipline

☐ Anthropology

☐ Policy, health policy, planning, political science

☐ Sociology

☐ Law

☐ CBO, nonprofit, center, neighborhood Assoc.

☐ Government agency (CDC, NIH, health department)

☐ Foundation, institute (Kellogg, RWJF, Kreiger, CA Endowment)

☐ Other academic discipline (edu, social work, nursing, urban planning, medicine)

☐ Other: ___________

**II. Research Design (check all that apply)**

☒ Case Study

☐ Evaluation

☐ Observational and cross-sectional

☐ Observational and longitudinal

☐ Natural experiment with 1 post measurement point

☐ Natural experiment with multiple measurement points

☐ Mixed methods

☐ Document review

☐ Marked for exclusion: surveillance data, contaminant sampling, no design mentioned, descriptive, summary report, review article

☐ no design reported

☐ Other: __________

**Guidance**

| | |
|---|---|
| **Case study** | A quantitative or qualitative method of inquiry of a particular instance or situation over time within its real-life context; often descriptive in nature and based on local knowledge. |
| **Evaluation** | Systematic collection of information, using methods and design strategies of traditional research with primary purpose of assessing characteristics, processes, strategies, and or outcomes related to a specific program or activity. |
| **Observational and cross-sectional** | Description of characteristics, analysis of data from one time point. |
| **Observational and longitudinal** | Several observations of the same people or groups (e.g., defined community, city) over a period of time; e.g. cohort, panel, repeated measure |
| **Mixed methods** | Research involves collecting and analyzing both quantitative and qualitative data |
| **Natural experiment** | Clearly defined exposure or intervention involving a well-defined subpopulation and includes a comparison group; also referred to as quasi-experimental |
| **Marked for exclusion** | Surveillance data, contaminant sampling, summary report, review article |

III. **Methods** (as named by the author; check all that apply)

Data Collection Methods

☐ Assessment (e.g. health, ecological, exposure, risk, monitoring)

☐ Focus group, group discussion

☐ Interviews

☐ Document review

☐ Other participatory methods

☐ Other data collection methods

☐ survey/ questionnaire

Research Orientation (explicit or implied)

☐ CBPR/PAR orientation (Community based participatory research/participatory action research)

**Guidance**

| | |
|---|---|
| **Interview** | In-depth conversation and exploration of participant experiences, feelings and attitudes about a particular subject matter; often structured and conducted under controlled conditions, or they may be conducted with a loose set of questions asked in an open-ended manner. |
| **Focus Group** | Group discussion hosted by a moderator in which participants discuss their ideas and insights in response to open-ended questions and share their perceptions, opinions, beliefs, and attitudes toward a particular subject matter. |
| **Observation** | Systematic description of events/situations that may involve informal interviewing, writing detailed field notes; process of learning through exposure or involvement in activities/experiences of the participants in a natural setting. |
| **Photovoice** | A participatory method that has community participants use photography, and stories about their photographs, to identify and represent issues of importance to them. |

**IV. System, Policy or Environmental change target** (check all that apply)

a. **Pollution concerns**

☐ Air pollution/air quality

☐ Illegal dumping

☐ Hazardous waste (brownfields, superfund, chemical contaminants, soil contaminants, fish contaminants)

☐ Water quality (drinking or groundwater)

**V. Community engagement & strategies for reducing environmental threats** (check all that apply)

**Capacity Building and advocacy strategies used for reducing pollution** (check all that apply for those explicitly)

☒ authentic participation process

☐ training & technology transfer

☐ technical assistance

☐ community organizing, mobilization

☐ partnership/coalition building

☐ media advocacy

☐ authentic participation (i.e., citizen science)

☐ CBPR, (i.e. lay health advisor model, lay health worker, community health worker, promotores)

☐ photovoice

☐ policy advocacy

☐ litigation, testimony, mediation, legal strategy, public hearing

☐ letter writing, petitions

☐ public protest, civil disobedience

☐ Other: ______________________________

**Guidance**

| | |
|---|---|
| ***Authentic participation processes*** | Agency designed participation processes that improve community capacity by getting people involved early, providing them with information and resources for full participation, and ensuring that outcomes reflect their participation (i.e. citizen science). |
| ***CBPR*** | A research process in which community residents participate in selecting issues, designing studies, interpreting findings, and presenting results to policymakers for the purpose of reducing environmental health inequities and promoting healthier public policies (i.e. utilization of the lay health advisor (LHA) model in which individuals of the community are trained as resources to assist in the education and distribution of materials for research studies) |
| ***Community organizing/social action*** | Community mobilization and organization to enable a disadvantaged segment of the population to make demands on the larger community for increased resources and more equitable policies. |
| ***Empowerment approaches*** | Process by which individuals, communities, and organizations gain power and mastery over their lives in the context of changing their social and political environment to improve equity and quality of life. |
| ***Technical assistance*** | Tailored support that enables community participants to gain information or skills to solve problems or to participate more effectively in decision-making processes. |
| ***Training and technology transfer*** | Process by which community participants gain knowledge, skills, competencies, or technologies that enable them to participate in assessing and remediating environmental hazards and participating in relevant policy deliberations. |

**Other Strategies**

| | |
|---|---|
| ***Civil Disobedience*** | The refusal to comply with certain laws or to pay taxes and fines, as a peaceful form of political protest, that often includes nonviolent techniques such as boycotting, picketing |
| ***Letter writing*** | An organized effort to coordinate as many people as possible to write to a decision maker (legislative or facility) asking them to take a particular action. |
| ***Litigation*** | The process of taking legal action to enforce of defend a legal right. |
| ***Media advocacy*** | Strategic use of traditional or social media outlets to disseminate information and promote policy initiatives. |
| ***Partnership coalition building*** | Engaging individuals, organizations and governmental partners in addressing community concerns; harnessing existing resources to develop a community approach to achieve results. |
| ***Photovoice*** | A participatory method that has community participants use photography, and stories about their photographs, to identify and represent issues of importance to them. |
| ***Policy advocacy*** | Analysis of the cause of the problem and development of policy-based solutions to create sustainable change. |

**Activities to Enhance Community capacity (explicitly stated; check at least one and all that apply)**

☐ ***citizen participation*** - offering incentives for participation; conduct outreach to uninvolved sectors of population; community activism; and providing residents with voice in making key decisions;

☐ ***community history*** - context and analysis of previous efforts, assisting residents to study and analyze previous health and environmental issues facing community; prepare reports aimed at community residents that develop such understanding relative to others;

☐ ***community power*** – empowerment; join coalitions for environmental health to enhance community strength; provide community with information so they can confront special interests effectively; support political reforms that level the playing field for those with less influence; provide scientific information that can be used in political arena

☐ ***community values*** - Articulate values, shared norms and standards that underlie public health efforts related to environment, social justice, and democracy; defend community values on health against disease promoting organizations

☐ ***critical reflection*** **-** identification of successes and limitations of actions, assisting community residents to analyze and reflect on successes and limitations of their actions to promote environmental health;

☐ ***leadership*** - Prepare environmental activists to be leaders; educate community leaders about environmental issues; create forums to bring formal and informal community leaders together to consider environmental health issues; assist with strategic planning and policy development

☐ ***resources*** - Serve as bridge between community and external resources (e.g., state health dept, foundations); assist participants to identify and develop local assets; contribute staff time to community investigations; build capacity for advocacy; assist in writing grants and working with funders to support community groups.

☐ ***networks*** **-** partnership; support and nurture local, regional, and national coalitions that bring together concerned citizens, environmental activists, scientists, health professionals, and others for environmental health promotion activities;

☐ ***sense of community*** - Support community events that build sense of identity; create safe spaces for community residents to discuss, analyze, and study environmental health issues; and

☐ ***skills*** **-** offering workshops and technical assistance on environmental health issues; creating opportunities for participants to exchange skills; assist efforts to link those with skills inside and outside community to those with needs.

**Guidance**

| | |
|---|---|
| ***Citizen participation*** | *Examples include:* Providing opportunity for community members to engage, better define, analyze and act on issues in their community; Community is actively involved in the identification of problems, solutions to the problems and actions to resolve the problems |
| ***Community history*** | *Examples include:* Understanding of current physical, social, psychological, economic and political dynamics with making a connection to the community's past; application of this understanding in the creation of strategies or community action; **Exclude** – description of community in background only |
| ***Community power*** | *Examples include:* Ability of a community to advocate for change; ability to resist; have control over decisions on planning, implementation, evaluation; |
| ***Community values*** | *Examples include:* Community priorities, standards, and motivations are clearly defined |
| ***Critical reflection*** | *Examples include:* Ability to identify contrasting perspectives/motivations related to the change efforts; understanding of desired changes within the larger social, political, historical contexts |
| ***Leadership*** | *Examples include:* Identification of individuals or a group that directs and encourage participation, strategic planning |
| ***Resources*** | *Examples include:* Providing access to funds or organizing opportunity for fundraising, grants, other traditional capital; identification of people with skills, sharing knowledge |
| ***Networks*** | *Examples include:* Informal or formal partnerships (i.e. community group, coalition, neighborhood association, organization) that bring people together to address their concerns and problems |
| ***Sense of community*** | *Examples include:* Residents have a heightened level of concern for community issues, share emotional connectedness; |

| | |
|---|---|
| ***Skills*** | *Examples include:* Learning specific technical skills to identify or assess community concerns; exchanging knowledge and skills to implement plans |

**Environmental Outcomes (check all that apply)**

☐ reduction in exposure to pollutant

☐ clean-up of pollution concern

☐ remediation of toxic waste

☐ increased regulation of $PM_{2.5}$

☐ reduction of indoor allergen (mold, infestation of rodents, mites, roaches etc.)

☐ none reported

☐ other: ______________________

**Policy related Outcomes related to Community Advocacy Efforts (check at least one and all that apply)**

☐ enforcement environmental law/regulation; review of conditional-use permit

☐ mitigation of concern (reduction of risk by taking some other action e.g. improvements in new construction)

☐ prevention of industrial development of noxious facility

☐ introduction of legislative resolution to address toxic emissions; new law, mandate, regulation or ordinance to prohibit pollution

☐ plaintiff settlement or successful litigation

☐ increased compliance or mandatory payment of fines for pollution/safety violations

☐application of any aforementioned effort with a failed outcome

☐ meeting with politician to discuss environmental concerns

☐ none reported

☐ other: ___________________

VI. **Use of Theoretical frameworks (check all that apply)**

**Author mentions using a theoretical framework, model, or pieces of theory combined**

☐ yes ☐ no

**Commonly used community theories (select only if named by the authors)**

☐ community capacity

☐ community empowerment

☐ efficacy

☐ EJ frame/perspective

☐ collective action

☐ transformative learning theory

☐ Bronfenbrenner's bioecological theory

☐ new social movement theory

☐ community mentoring model

☐ democratic public theory

☐ vulnerability theory

☐ empowerment education theory

☐ capability theory

☐ implementation theory

☐ social support theory

☐ theory of resilience

☐ theory of planned behavior

☐ grounded theory

☐ Other: ________________________

**Application of theoretical framework (as described by the author)**

☐ Informed data collection instrument

☐ Informed sampling methods

☐ Constructs used for analysis (e.g. provided framework)

☐ Developed as a result of analysis (e.g. grounded theory used with qualitative work)

☐ Mentioned in intro/discussion but not operationalized or measured

☐ No mention of guiding theory

☐ Other application:

**Guidance**

For the purposes of this review, a theory is defined as a set of interrelated concepts, definitions, or propositions presented to describe a particular orientation for understanding a problem, explaining observations, practices, experiences and/or behavior. Specifically, the term construct is defined as a major concept, component, building block, primary or core element used to understand a theory.

**VII. Other areas of interest:**

Is this research relevant to climate change? ☐ yes ☐ no

If there is no direct policy outcome, is there mention of a policy implication of the study? ☐ yes ☐ no

**Table S4.** Examples of General Capacity-building and Community change Strategies.

| | | |
|---|---|---|
| ***Letter writing*** | *An organized effort to coordinate as many people as possible to write to a decision maker (legislative or facility) asking them to take a particular action* | A community health department approach was used to improve child asthma-related health concerns and reduce environmental asthma triggers. Intervention strategies focused on community organizing, among residents who disproportionately experienced heavy vehicle pollution, to affect city policy. Activities entailed door-to-door canvassing; distribution of fact sheets; writing and distribution of press releases; policy briefings with local representatives; presentation at conferences, community meetings, and forums; collecting signatures; and letter writing campaigns [47]. |
| ***Media Advocacy*** | *Strategic use of traditional or social media outlets to disseminate information and promote policy initiatives.* | A project partnership comprised of a nonprofit research organization, an institute, and a neighborhood improvement initiative, collaborated to identify indicators, collect data, and summarized findings to compare outcomes to the broader city and state. The partnership released a report with recommendation to the local media detailing findings that residents were being exposed to 5 times more diesel particles than other parts of the city and children suffered asthma rates that were 7 times the states average. Community town hall meetings and forums were held to develop a proposal for a city ordinance outlining an alternate diesel truck route that was unanimously passed by the City Council [68]. |
| ***Lay Health Advisor model*** | *Utilization of community individuals who often share similar social, economic, cultural and linguistic backgrounds, and values as the study population to assist with study activities, mobilize resources, and engage with study population.* | A university-community partnership actively trained 17 *promotoras de salud* (lay health promoters) as co-researchers and policy change advocates on topics including land use and environmental health. *Promotoras* participated in door-to-door canvassing to collect survey data, participated in the translation of research findings, involvement in the interpretation, and recommendations for action. *Promotoras* also led community meetings to further disseminate the study findings and elicit additional input [69]. |
| ***Litigation*** | *The process of taking legal action to enforce of defend a legal right* | Approaches used by Washington coastal communities successfully challenged the construction of what would have been the largest coal terminal in North America by creating a partnership uniting the sovereign Lummi Nation, faith-based, and environmental groups. Tribes cited treaty-fishing rights and environmental organizations believed that such export activities and facilities posed a risk to public and environmental health. The partnership sued industry for illegal land clearing and damage to wetlands, followed by the gathering of 10,000 signatures from voters in the city, and a joint campaign producing up to 25,000 letters to Congress addressing the threats to Lummi fishing rights [70]. |
| ***Civil Disobedience*** | *The refusal to comply with certain laws or to pay taxes and fines, as a peaceful form of political protest, that often includes nonviolent techniques such as boycotting, picketing.* | As a result of an increased presence of mountaintop coal removal, nearby Appalachian communities have experienced frequency of coal trucks creating pollution, noise, and endangering other motorists in neighboring communities. A neighboring women's group engaged in various forms of social protest including direct action of blocking roads from passing trucks, organizing rallies, educational campaigns, monitoring coal industry meetings, and lobbying state government advocating for coal field communities [71]. |
| ***Training*** | *Gaining skills and knowledge to address environmental concerns.* | Local collaborative activism spearheaded the development of a USEPA community environmental justice program initiative to "help alleviate environmental and health challenges" in communities of color facing toxic pollution. Funding contributed to the development job training for 100 families in the community about emergency response and chemical cleanout training for community representatives. Additional education was provided by USEPA staff on state air quality initiatives, drinking water, and trained 32 teachers on how to properly dispose of chemicals in the classroom [72]. |
| ***Citizen Science*** | *Research that is conducted in whole or part by nonscientists and members of the general* | Targeted strategies were developed to address high rates of asthma and other respiratory diseases in the community by better understanding the air pollution profile within its neighborhood borders. An Air Sensor Toolbox for Citizen Scientists |

| | | |
|---|---|---|
| | *public, often in collaboration with or under direction of professional scientists and scientific institutions.* | was created to help with the collection, analysis, interpretation, and communication of effective air quality data. Sensors were placed at homes near sources experiencing increased air pollution for seven continuous days. The project successfully engaged residents in air quality measurement data collection that helped to build capacity and knowledge for future air quality projects [73]. |
| ***Partnership/ Community Advisory Board*** | *Engaging individuals, organizations and governmental partners in addressing community concerns; harnessing existing resources to develop a community approach to achieve results.* | A university-tribal partnership was created among local tribes, residing in one of the world's largest lead and zinc mining areas, to address the elevated blood lead levels in Native American children resulting from the use of mine tailings in building and road construction. A community advisory board (CAB) was established to guide the project, comprised of members representing each of eight tribal communities, local public health, and environmental agencies that quarterly throughout the duration of the project. Forty natural helpers were recruited and trained on lead poisoning prevention strategies, planned community awareness and advocacy activities, and educated individuals in their social networks about lead [74]. |
| ***Technical Assistance*** | *Tailored support that enables participants to gain skills to problem solve or more effectively participate in decision-making; e.g. survey design, monitoring pollutants.* | Technical assistance (TA) was provided by statewide experts in asthma prevention and management, policy advocacy, and media and communication to a network of community organizations working to reduce community environmental asthma risk factors. Skill building trainings were provided in data collection and analysis, community education methods focused on policy, fundraising, and outreach strategies. Additional TA entailed helping assess local and state level environmental inequities, use of data to support a policy position, preparing testimony and evidence, grant writing skills and outreach strategies, and learning policy forums and procedures at the local, regional, and state levels [75]. |
| ***Policy Advocacy*** | *Analysis of the cause of the problem and development of policy-based solutions to create sustainable change.* | Technical assistance was requested from an environmental health research and advocacy organization to assess biomarkers of exposure susceptibility among Yupik residents living near a former military base. The research conducted found blood serum in residents to have elevated levels of polychlorinated biphenyls (PCBs) with higher levels among those living near a military site. Study findings were shared with the US Army Corp of Engineers and residents prompted them to establish a Restoration Advisory Board providing residents with opportunity to comment and advise in the remediation process. The advocacy of this board resulted in the designation of the site as one of one of the highest priorities for clean-up in Alaska and allocation of $100 million remediation funds [76]. |
| ***Photovoice*** | *A participatory method that has community participants use photography, and stories about their photographs, to identify and represent issues of importance to them.* | An ethnographic study was conducted among Mexican American farmworker families living in communities with high rates of asthma, to explore the relationship of childhood asthma, pesticide exposure, and aerial spraying. Photovoice methodology was employed to allow mother-child dyads to dialogue and share knowledge about personal/community issues, record and reflect on community strengths and concerns, and advocate for policy change with policymakers. Prior to photography, participants were asked to identify barriers and facilitators of health and places and things that contribute to asthma and help or improve asthma symptoms. After photography, participants reflected on causes and solutions to the problem through the "SHOWeD" process: S- what do you see happening; H- what is really happening; O- how does this related to our lives; W- why does this problem/asset exist?; and D- what can we do about it? Final results were displayed through individual posters and discussed among participants [77]. |

**Table S5.** Activities to Enhance Dimensions of Community Capacity by Study.

| | | Dimensions of Community Capacity | | | | | | | | | |
|---|---|---|---|---|---|---|---|---|---|---|---|
| | | **Citizen participation** | **Community history** | **Community power** | **Community values** | **Critical reflection** | **Leadership** | **Networks** | **Resources** | **Sense of community** | **Skills** |
| **N (%)** | **Year** | **53 (96.4%)** | **22 (37.9%)** | **45 (77.6%)** | **11 (18.9%)** | **13 (22.4%)** | **45 (77.6%)** | **47 (81.0%)** | **32 (58.2%)** | **36 (62.1%)** | **12 (20.7%)** |
| Bullard, R. D. and B. H. Wright. The quest for environmental equity - mobilizing the African-American community for social-change. Soc & Nat Resources 3(4): 301-311. | **1990** | **X** | | **X** | | | **X** | **X** | | | |
| Capek, S. M. Environmental justice regulation and the local community. Int J Health Serv 22(4): 729-746. | **1992** | **X** | | **X** | **X** | | **X** | | | | |
| Jacobs, J. A community organizing case study: an analysis of cap-it's strategy to prevent the location of a toxic waste incinerator in their community. Int Q Community Health Educ 13(3): 253-263. | **1992** | **X** | | | **X** | | **X** | **X** | | | |
| Čapek, S. M. The environmental justice frame: A conceptual discussion and an application. Soc Problems 40(1): 5-24. | **1993** | **X** | | **X** | | | **X** | **X** | **X** | | |
| Brown, P. and S. Masterson-Allen. The toxic-waste movement - A new-type of activism. Soc Nat Resour 7(3): 269-287. | **1994** | **X** | | **X** | | | **X** | **X** | | | **X** |
| Allen, B. L. Saving St. Gabriel: The emergence of a new African-American town. Contemporary Justice Review 4(2): 145. | **2001** | **X** | **X** | **X** | **X** | **X** | **X** | **X** | **X** | **X** | |
| Corburn, J. Combining community-based research and local knowledge to confront asthma and subsistence-fishing | **2002** | **X** | | | | | **X** | **X** | **X** | **X** | **X** |

| | | | | | | | | | | | |
|---|---|---|---|---|---|---|---|---|---|---|---|
| hazards in Greenpoint/Williamsburg, Brooklyn, NY. Environ Health Perspect. S110-2: 241-8. | | | | | | | | | | | |
| Green, L., et al. "Hey, mom, thanks!": Use of focus groups in the development of place-specific materials for a community environmental action campaign. Environ Health Perspect 110 Suppl 2: 265-269. | **2002** | X | | | | | X | | | | |
| Loh, P., et al. "From Asthma to AirBeat". Environ Health Perspect S110: 297. | **2002** | X | X | X | X | X | X | | X | X | |
| Brown, P., et al. The health politics of asthma: environmental justice and collective illness experience in the United States. Soc Sci Med 57(3): 453-464. | **2003** | X | | X | | | X | X | | | X |
| Johnson, G. S. Grassroots activism in Louisiana. Humanity & Soc 29(3/4): 285-304. | **2005** | | | | | | X | X | X | | |
| Blodgett, A. D. An analysis of pollution and community advocacy in 'Cancer Alley': setting an example for the environmental justice movement in St James Parish, Louisiana. Local Environ 11(6): 647-661. | **2006** | X | | X | | | X | | X | | |
| Grineski, S. E. Local Struggles for Environmental Justice: Activating Knowledge for Change. J Poverty 10(3): 25-49. | **2006** | X | | X | | | X | X | X | X | |
| Tajik, M. and M. Minkler. Environmental justice research and action: a case study in political economy and community-academic | **2006** | X | | X | | X | X | X | X | X | |

| | | | | | | | | | | | |
|---|---|---|---|---|---|---|---|---|---|---|---|
| collaboration. Int Q Community Health Educ 26(3): 213-231. | | | | | | | | | | | |
| Higman, K., et al. Using the PACE EH model to mobilize communities to address local environmental health issues - A case study in Island county, Washington. J Environ Health 70(1): 37-41. | **2007** | x | | x | | | x | x | x | | |
| Wilson, S. M., et al. Use of EPA collaborative problem-solving model to obtain environmental justice in North Carolina. Prog Community Health Partnersh. 1(4): 327-337. | **2007** | x | | x | | | x | x | x | x | |
| Barry, J. M. A small group of thoughtful, committed citizens: Women's activism, environmental justice, and the Coal River Mountain watch. Environ Justice 1(1): 25-33. | **2008** | x | | x | x | | x | | | | x |
| Drury, R. T. Moving a Mountain: The struggle for environmental justice in southeast Los Angeles. Environ Law Reporter: News & Analysis 38(5): 10338-10346. | **2008** | x | | x | | x | x | x | x | | |
| Senier, L., et al. Brown superfund basic research program: A multi-stakeholder partnership addresses real-world problems in contaminated communities. Environ Sci Tech 42(13): 4655-4662. | **2008** | x | | x | | | x | x | x | x | |
| Sherman, D. J. Disruption or convention? A process-based explanation of divergent repertoires of contention among opponents to low-level radioactive | **2008** | | x | x | | | | x | x | | x |

| | | | | | | | | | | | |
|---|---|---|---|---|---|---|---|---|---|---|---|
| waste disposal sites. Soc Movement Studies 7(3): 265-280. | | | | | | | | | | | |
| Sullivan, J. and J. Parras. Environmental justice and Augusto Boal's theatre of the oppressed: a unique community tool for outreach, communication, education and advocacy. Theory in Action 1(2): 20-39. | **2008** | x | x | x | x | | | x | x | x | x |
| Wing, S., et al. Integrating epidemiology, education, and organizing for environmental justice: community health effects of industrial hog operations. Am J Public Health 98(8): 1390-97. | **2008** | x | x | x | | | | x | | x | |
| Schelly, D. and P. B. Stretesky. An analysis of the path of least resistance argument in three environmental justice success cases. Soc Nat Resour 22(4): 369-380. | **2009** | | x | | | | x | x | | | |
| Wier, M., et al. Health, traffic, and environmental justice: collaborative research and community action in San Francisco, California. Am J Public Health 99 Suppl 3: S499-504. | **2009** | x | x | x | | | x | x | | x | |
| Williams, E. M., et al. Behind the fence forum theater: an arts performance partnership to address lupus and environmental justice. New Solut 19(4): 467-479. | **2009** | x | x | x | | | x | x | x | x | x |
| Emmett, E. A. and C. Desai. Community first communication: reversing information disparities to achieve environmental justice. Environ Justice 3(3): 79-84. | **2010** | x | | x | | | | x | x | | |

| | | | | | | | | | | | |
|---|---|---|---|---|---|---|---|---|---|---|---|
| Kegler, M. C., et al. Primary prevention of lead poisoning in rural Native American children behavioral outcomes from a community-based intervention in a former mining region. Fam Community Health 33(1): 32-43. | **2010** | X | | X | | | X | X | | X | |
| Minkler, M. Linking science and policy through community-based participatory research to study and address health disparities. Am J of Public Health 100: S81-7. | **2010** | X | | X | | X | X | X | X | X | |
| Minkler, M., et al. Si se puede: Using participatory research to promote environmental justice in a Latino community in San Diego, California. J Urban Health 87(5): 796-812. | **2010** | X | X | X | | X | X | X | X | X | |
| Parker, N. E. A., et al. (2010). Community organizing network for environmental health: using a community health development approach to increase community capacity around reduction of environmental triggers. J Prim Prev 31(1-2): 41-58. | **2010** | X | X | X | X | X | X | X | X | X | X |
| Sicotte, D. (2010). Don't waste us: Environmental justice through community participation in urban planning. Enviro Justice (19394071) 3(1): 7-11. | **2010** | X | X | X | | X | | X | | | |
| Gonzalez, P. A., et al. (2011). Community-based participatory research and policy advocacy to reduce diesel exposure in West Oakland, California. Am J of Public Health 101: S166-75. | **2011** | X | | X | | | X | X | | X | |

| | | | | | | | | | | | |
|---|---|---|---|---|---|---|---|---|---|---|---|
| Haynes, E. N., et al. (2011). Developing a bidirectional academic-community partnership with an Appalachian-American community for environmental health research and risk communication. Environ Health Perspect 119(10): 1364-1372. | **2011** | X | X | X | X | | X | X | X | | |
| Kreger, M., et al. (2011). Creating an environmental justice framework for policy change in childhood asthma: A grassroots to treetops approach. Am J Public Health 101 Suppl 1: S208-216. | **2011** | X | X | X | | X | | X | X | X | |
| Stedman-Smith, M., et al. (2012). Photovoice in the Red River Basin of the north: a systematic evaluation of a community-academic partnership. Health Promot Pract 13(5): 599-607. | **2012** | X | X | X | | | | X | | X | X |
| Dressel, A., et al. The westlawn partnership for a healthier environment: promoting environmental justice and building community capacity. Environ Justice, 6(4): 127-132. | **2013** | | | X | | | X | X | X | X | |
| Garcia, A. P., et al. THE (Trade, Health, Environment) impact project: a community-based participatory research environmental justice case study. Environ Justice 6(1): 17-26. | **2013** | X | X | X | | | X | X | X | X | |
| Balazs, C. L. and R. Morello-Frosch. The three Rs: how community-based participatory research strengthens the rigor, relevance, and reach of science. Environ Justice, 6(1): 9-16. | **2013** | X | | X | | | | | X | X | |

| | | | | | | | | | | | |
|---|---|---|---|---|---|---|---|---|---|---|---|
| Miller, P. K., et al. Community-based participatory research projects and policy engagement to protect environmental health on St Lawrence Island, Alaska. Int J Circumpolar Health 72. | **2013** | X | X | X | | | X | X | X | X | |
| Sadd, J., et al. The truth, the whole truth, and nothing but the ground-truth: methods to advance environmental justice and researcher-community partnerships. Health Educ Behav 41(3): 281-290. | **2014** | X | | X | | | X | | | X | |
| Bell, S. E. Bridging activism and the academy: Exposing environmental injustices through the feminist ethnographic method of photovoice. Human Ecol Rev 21(1): 27-58. | **2015** | X | | X | | | X | | | | X |
| Hines, R. I. The price of pollution: The struggle for environmental justice in Mossville, Louisiana. Western J Black Studies 39(3): 198-208. | **2015** | X | X | X | | | X | X | X | X | |
| Jiao, Y., et al. Application of citizen science risk communication tools in a vulnerable urban community. Int J Environ Res Public Health 13(1): ijerph13010011. | **2015** | X | | | | | X | X | | X | |
| Rohlman, D., et al. A community-based approach to developing a mobile device for measuring ambient air exposure, location, and respiratory health. Environ Justice 8(4): 126-134. | **2015** | X | | | X | | | | | | |
| Schwartz, N. A., et al. Where they (live, work and) spray: pesticide | **2015** | X | | X | | | X | X | | X | |

| | | | | | | | | | | | |
|---|---|---|---|---|---|---|---|---|---|---|---|
| exposure, childhood asthma and environmental justice among Mexican-American farmworkers. Health Place 32: 83-92. | | | | | | | | | | | |
| White, B. M. and E. S. Hall. Perceptions of environmental health risks among residents in the Toxic Doughnut: opportunities for risk screening and community mobilization. BMC Public Health 15: 1230. | **2015** | X | X | X | | | | X | | | |
| Cohen, A. K., et al. Surveying for environmental health justice: community organizing applications of community-based participatory research. Environ Justice 9(5): 129-136. | **2016** | X | | | | | X | X | X | X | |
| Kaup, B. Z. and D. Casey. Coalition of injustice? Bodies, business, and the biosphere in struggles against unwanted land uses. Environ Politics 25(3): 494-512. | **2016** | X | X | | | X | X | X | | | |
| Robinson, E. E. Sharing Stories. Humanity Soc 40(4): 442-461. | **2016** | X | | X | X | | | | X | X | |
| Spencer-Hwang, R., et al. Strategic partnerships for change in an environmental justice community: The ENRRICH study. Prog Community Health Partnersh. 10(4): 541-550. | **2016** | X | | | | | X | X | X | X | |
| Allen, M., et al. Stronger together: Strategies to protect local sovereignty, ecosystems, and place-based communities from the global fossil fuel trade. Marine Policy 80: 168-176. | **2017** | X | X | X | X | X | X | X | X | X | X |

| | | | | | | | | | | | |
|---|---|---|---|---|---|---|---|---|---|---|---|
| Brickle, M. B. and R. Evans-Agnew. Photovoice and youth empowerment in environmental justice research: A pilot study examining woodsmoke pollution in a pacific northwest community. J Community Health Nurs. 34(2): 89-101. | **2017** | X | | X | | X | X | X | | X | X |
| Dhillon, C. M. Using citizen science in environmental justice: participation and decision-making in a Southern California waste facility siting conflict. Local Environ 22(12): 1479-1496. | **2017** | X | | X | | | X | X | X | X | |
| Kennedy, A., et al. "Taking away David's sling": Environmental justice and land-use conflict in extractive resource development. Local Environ 22(8): 952-968. | **2017** | | X | | | | X | | | | |
| Kaufman, A., et al. A citizen science and government collaboration: Developing tools to facilitate community air monitoring. Environ Justice 10(2): 51-61. | **2017** | X | | | | | | X | | X | |
| Sanchez, H. K., et al. Confronting power and environmental injustice: legacy pollution and the timber industry in Southern Mississippi. Soc Nat Resour 30(3): 347-361. | **2017** | X | X | X | | X | | X | | X | |
| Wilson, S., et al. Soil contamination in urban communities impacted by industrial pollution and goods movement activities. Environ Justice 10(1): 16-22. | **2017** | X | | X | | | X | X | | X | |

| | | | | | | | | | | | |
|---|---|---|---|---|---|---|---|---|---|---|---|
| Bruno, T. and W. Jepson. Marketisation of environmental justice: U.S. EPA environmental justice showcase communities project in Port Arthur, Texas. Local Environ 23(3): 276-292. | **2018** | X | | X | | | X | X | X | X | X |
